# Pharmacological modulation of aversive responsiveness in honey bees

**DOI:** 10.3389/fnbeh.2013.00221

**Published:** 2014-01-07

**Authors:** Stevanus R. Tedjakumala, Margaux Aimable, Martin Giurfa

**Affiliations:** ^1^Centre National de la Recherche Scientifique (CNRS), Research Center on Animal Cognition (UMR5169)Toulouse, France; ^2^University Paul-Sabatier, Research Center on Animal Cognition (UMR5169)Toulouse, France

**Keywords:** neuromodulation, honey bee, sting extension response (SER), aversive responsiveness, octopamine, dopamine, serotonin, 20-hydroxyecdisone

## Abstract

Within a honey bee colony, individuals performing different tasks exhibit different sensitivities to noxious stimuli. Noxious-stimulus sensitivity can be quantified in harnessed bees by measuring the sting extension response (SER) to a series of increasing voltages. Biogenic amines play a crucial role in the control of insect responsiveness. Whether or not these neurotransmitters affect the central control of aversive responsiveness, and more specifically of electric-shock responsiveness, remains unknown. Here we studied the involvement of the biogenic amines octopamine, dopamine and serotonin, and of the ecdysteroid 20-hydroxyecdisone in the central control of sting responsiveness to electric shocks. We injected pharmacological antagonists of these signaling pathways into the brain of harnessed bees and determined the effect of blocking these different forms of neurotransmission on shock responsiveness. We found that both octopamine and 20-hydroxyecdisone are dispensable for shock responsiveness while dopamine and serotonin act as down-regulators of sting responsiveness. As a consequence, antagonists of these two biogenic amines induce an increase in shock responsiveness to shocks of intermediate voltage; serotonin, can also increase non-specific responsiveness. We suggest that different classes of dopaminergic neurons exist in the bee brain and we define at least two categories: an instructive class mediating aversive labeling of conditioned stimuli in associative learning, and a global gain-control class which down-regulates responsiveness upon perception of noxious stimuli. Serotonergic signaling together with down-regulating dopaminergic signaling may play an essential role in attentional processes by suppressing responses to irrelevant, non-predictive stimuli, thereby allowing efficient behavioral performances.

## Introduction

Honey bees are a well-established model for the study of learning and memory (Menzel, 1999; Giurfa, 2007). In the laboratory, associative olfactory learning is studied using harnessed bees subjected to Pavlovian protocols such as the appetitive conditioning of the proboscis extension reflex (PER) (Takeda, 1961; Bitterman et al., 1983; Giurfa and Sandoz, 2012) and the aversive conditioning of the sting extension reflex (SER)(Vergoz et al., 2007; Carcaud et al., 2009; Giurfa et al., 2009). In the former, bees learn to associate an odorant as conditioned stimulus (CS) with sucrose solution as unconditioned stimulus (US). In the latter, bees learn the association between an odorant as CS and an electric shock as US. Although much is known about PER conditioning in terms of underlying circuitries, neural structures and neurotransmitters (Menzel, 1999; Giurfa, 2007; Giurfa and Sandoz, 2012), less is known about SER conditioning given its recent establishment (Vergoz et al., 2007; Tedjakumala and Giurfa, 2013).

A proper characterization of the SER protocol implies a thorough analysis of the unconditioned response, the sting extension response. This response is elicited by noxious stimuli (Breed et al., 2004) and can be systematically triggered in harnessed bees by the delivery of a mild electric shock (Núñez et al., 1983, 1997; Lenoir et al., 2006; Vergoz et al., 2007). Sting responsiveness to shocks varies among bees within a colony (Lenoir et al., 2006; Roussel et al., 2009). For instance, foragers exhibit higher sting extension responsiveness than guards when stimulated with a series of increasing voltages. Based on this different sensitivity, they also learn better odor-shock associations (Roussel et al., 2009). These results demonstrate the crucial role of US sensitivity for learning and retention performances as underlined by models of classical conditioning, where US salience directly affects learning rate (Rescorla and Wagner, 1972). They also show that sensitivity to noxious stimulations may determine behavioral biases and specializations within the hive, thus contributing to the social organization of the colony (Roussel et al., 2009; Tedjakumala and Giurfa, 2013).

Biogenic amines play a crucial role in the control of insect responsiveness. Unconditioned appetitive responsiveness, measured through PER to a series of increase concentrations of sucrose solution (Pankiw and Page, 1999, 2000, 2003; Scheiner et al., 2004), is modulated by octopamine (OA) and dopamine (DA) signaling (Scheiner et al., 2002). For instance, feeding or injection of both OA and tyramine, an OA precursor, significantly increase PER to sucrose stimulation (Scheiner et al., 2002); on the contrary, DA decreases sucrose responsiveness when injected into the thorax but has no effect if fed. Consistently, injection or feeding of the DA receptor agonist 2-amino-6,7-dihydroxy-1,2,3,4-tetrahydronaphthalene (6,7-ADTN) reduces sucrose responsiveness significantly (Scheiner et al., 2002). Whether or not these biogenic amines affect unconditioned *aversive* responsiveness remains unknown. In particular, the implication of these neurotransmitters in the control of the *unconditioned SER* (i.e., in electric-shock responsiveness) has not been studied until now. In isolated abdominal preparations, OA potentiates reflexive sting extension responses and this potentiation persists for at least 3 h (Burrell and Smith, 1995). Yet this analysis does not reveal how aminergic signaling at the brain level drives sting responsiveness and the perception of noxious stimulations.

Studies on olfactory SER conditioning have shown that DA and the ecdysteroid 20-hydroxyecdisone (20E) are differently involved in this form of aversive learning (Vergoz et al., 2007; Geddes et al., 2013). DA is thought to mediate the aversive properties of electric shock as blocking of DA signaling impairs aversive learning and retention (Vergoz et al., 2007). 20E increases the expression of the DA receptor gene, Amdop2, and reduces the expression of the putative dopamine/ecdysone receptor gene, Amgpcr19, that tends to be highly expressed in the brains of foragers exhibiting strong aversive learning (Geddes et al., 2013); as a consequence, higher levels of 20E correlate with deficient aversive learning performances (Geddes et al., 2013). Serotonin (5-HT) has been repeatedly related to aggressiveness in invertebrates (Kravitz and Huber, 2003) and SER is a component of aggressive/defensive behaviors (Breed et al., 2004); however, the potential role of 5-HT in aversive responsiveness has not been addressed until now. Here we studied the involvement of the biogenic amines OA, DA and 5-HT, and of the ecdysteroid 20E in sting responsiveness to electric shocks of increasing voltage. We used pharmacological blocking procedures to determine if and how these different neural signaling pathways affect shock responsiveness in honey bees. As variations in shock responsiveness correlate with diverse behavioral specializations within the colony (Roussel et al., 2009; Tedjakumala and Giurfa, 2013), our experiments allow discussing the functions of biogenic amines for the social organization of the hive.

## Materials and methods

### Insects

Honey bees, *Apis mellifera*, were obtained from outdoor colonies. Nectar foragers were collected twice a day, between experimental series, from an artificial feeder to which they were previously trained. The feeder was located at 20 m from the hive and contained sucrose solution 40% (weight/weight). Nectar foragers were used because of their higher shock responsiveness (Roussel et al., 2009) and to reduce the high variability in biogenic amine titers that exist between different castes within a colony (Wagener-Hulme et al., 1999). We aimed, in this way, at ensuring that pharmacological treatments act on comparable levels.

**Figure 1:**
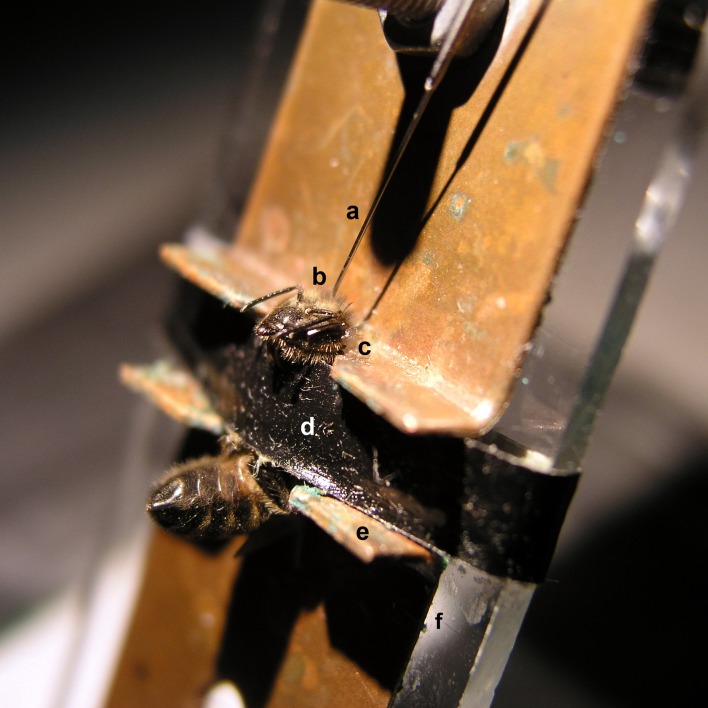
**Brain injection via the ocellar tract in a honey bee harnessed on a shock delivery setup.** A tiny hole was pricked into the cornea of the median ocellus to allow the insertion of a Hamilton syringe **(a)** located above the bee. The syringe allows delivery of the drug to be tested in the median ocellar tract **(b)**, which runs medially and caudally from the dorsal margin of the head capsule into the protocerebrum. The head of the bee is fixed to the metallic plate by means of a low-temperature melting wax **(c)** to reduce movements during injection. A girdle **(d)** is used to clamp the thorax to restrain mobility during the experiment. The bee acts as a bridge between the two metallic plates **(e)** fixed on a Plexiglas plate **(f)**. EEG cream was smeared on the two notches of the metallic plates to ensure good contact between the plates and the bee. The bee closes a circuit and receives a 2-s mild electric shock which induces the sting extension reflex (SER).

Once captured, the bees were brought to the laboratory and chilled on ice for 5 min until they stopped moving. They were then harnessed on individual holders (Figure 1) designed for aversive stimulation via delivery of an electric shock (Vergoz et al., 2007; Carcaud et al., 2009; Giurfa et al., 2009). Holders consisted of two brass plates fixed to a Plexiglas plate. Brass plates were connected to the output of the stimulator (60 Hz—AC current). The resistance measured between the two plates in the presence of the bee was 200–300 KΩ. Conductance gel was applied below the thorax to ensure efficient shock delivery. Low melting-point wax was used to immobilize the head and facilitate drug injection. Once fixed, each bee was fed with a droplet (5 μl) of sucrose solution 30% and kept resting for 1.5 h.

### Measuring shock responsiveness

Responsiveness to electric shock was measured using the SER. We stimulated bees with increasing voltages and recorded whether the bee extended its sting. The following voltages were applied in ascending order: 0.25, 0.5, 1, 2, 4, and 8 V. By alternating between a non-shocked (placement) and a shocked phase every 5 min, each individual was given, in this way, a 10-min shock interval. Each shock trial lasted 20 s; it consisted of 10 s of familiarization in the setup, followed by 2 s of electric shock; afterwards, the bee stayed for another 8 s before being replaced by the next test subject. Placement trials, in which the bee was placed in the setup during 20 s without shock delivery, were interspersed between shock trials to avoid sensitization. The inter-stimulus interval was approximately 1 min.

As shock responsiveness can vary from day to day depending on weather and/or intracolonial conditions, the response of experimental groups was always measured in parallel with that of their corresponding control groups.

### Pharmacological drugs and injections

A tiny hole was pricked into the cornea of the median ocellus to allow the insertion of a 10 μl-syringe (World Precision Instrument), which was used to inject 200 nl of each drug solution. Drugs were injected into the brain of immobilized bees along the median ocellar nerve (Figure 1). The ocellar nerve consists of a thick fiber bundle, approximately 40 μm in diameter, which runs medially and caudally from the dorsal margin of the head capsule into a depth of 300 μm into the protocerebrum. Previous works have shown that drugs migrate through the ocellar tract into the bee brain and that drug distribution is fast (less than 5 min) and homogenous within the brain (Menzel et al., 1999). After use, syringes were cleaned in PBS, ethanol and distilled water, completing three full wash cycles in each case.

The following substances, were injected 30 min before the experiment: *epinastine hydrochloride* [OA receptor antagonist (Roeder et al., 1998)], *cis-(Z)-flupentixol dihydrochloride* [DA receptor antagonist (Blenau et al., 1998)], *methiothepin mesylate* [5-HT antagonist (Blenau and Thamm, 2011)], *cyproheptadine hydrochloride sesquihydrate* [5-HT antagonist; (Howarth et al., 2002)], *ketanserin* [5-HT antagonist; (Wedemeyer et al., 1992; Howarth et al., 2002)], *20E* [ecdysteroid; (Geddes et al., 2013)], and PBS (control). Three different drugs were thus used to study the role of 5-HT in aversive responsiveness, which had never been addressed until now.

All substances, except for 20E and PBS, were obtained from Sigma-Aldrich France. 20E was kindly provided by Dr. Rodrigo Velarde (Wake Forest University, Winston-Salem, USA); PBS was obtained from EUROMEDEX (Strasbourg, France). Injection time was chosen based on previous experiments which have shown that the effects of aminergic blockers reach a stable level approximately 30 min after drug application (Mercer and Erber, 1983; Blenau and Erber, 1998; Scheiner et al., 2002; Vergoz et al., 2007).

20E was first dissolved in 1 ml isopropanol 100% to prevent crystallization of the steroid, resulting in a stock solution of 10 mg/ml, which was then diluted down to 1 mg/ml in PBS. For all other substances, 1 mg was diluted in 1 ml PBS. Final concentrations obtained were 3.5 mM of epinastine, 1.97 mM of flupentixol, 2.21 mM of methiothepin, 2.85 mM of cyproheptadine, 1.83 mM for ketanserin and 2.08 mM of 20E. To test for dose-response effects, we prepared for each drug, except for cyproheptadine, two additional dilution series of 1:100 and 1:10000; for cyproheptadine only the additional dilution of 1:100 was used. In all case, aliquots were made and kept in −20°C until use. Each aliquot was used for one whole week and kept during this time in 4°C.

### Data analysis

The occurrence of SER was recorded during the 2 s of electric stimulation in shock trials, and during the corresponding 2 s without stimulation in placement trials. An observable sting extension was given a score of 1; incomplete sting movements were scored as 0. Sting responsiveness (% of bees responding to a given voltage) was then calculated. Two-Way ANOVA (Statistica, StatSoft) was used to compare each treatment against its PBS control and for inter-treatment comparisons. ANOVA procedures are applicable in the case of binary response variables despite their lack of normality if comparisons imply equal cell frequencies and at least 40 degrees of freedom of the error term (Lunney, 1970; Matsumoto et al., 2012), conditions which were fulfilled by our experiments. Under these conditions, the use of repeated-measurement ANOVA allowed, not only within-group analysis, but also between-group comparisons. An alpha level of 0.05 was used throughout.

## Results

### Effects of OA blocking on aversive responsiveness

**Figure 2:**
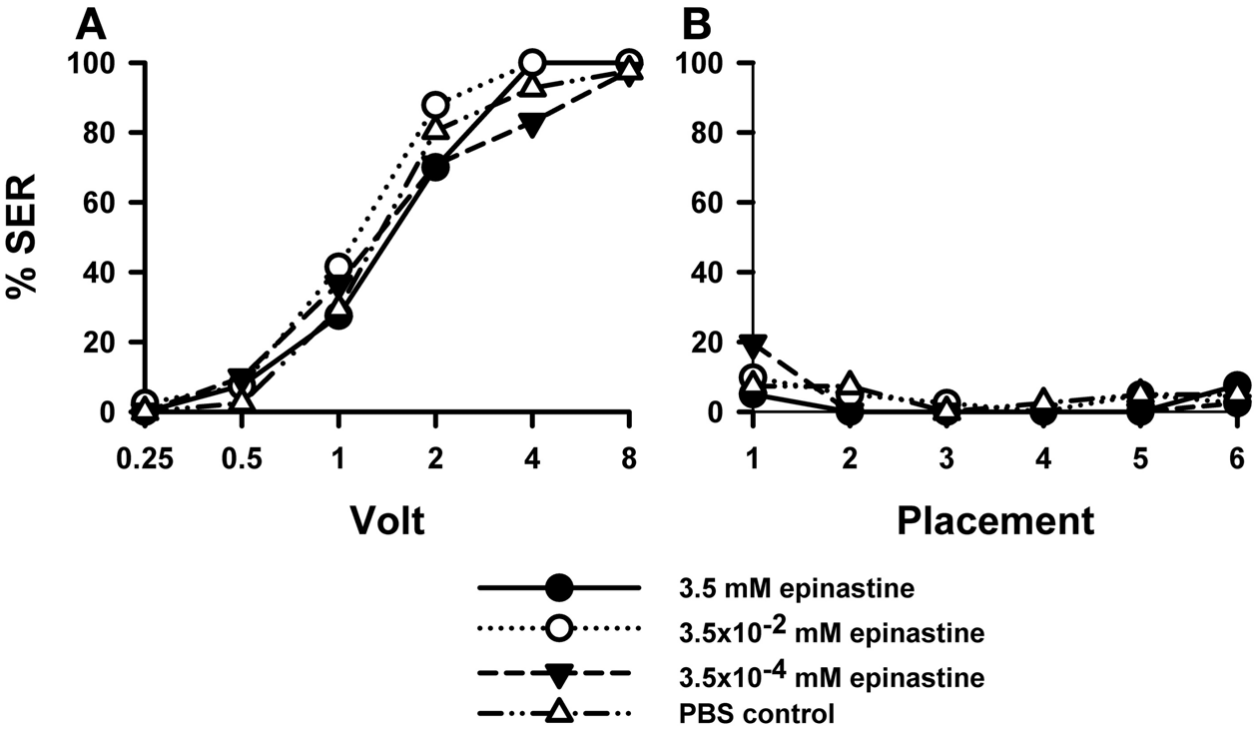
**Effects of OA blocking on aversive responsiveness.** Three different groups of bees were injected with three different concentrations of the OA antagonist epinastine (3.5 mM: *n* = 40; 3.5 × 10^−2^ mM: *n* = 41; 3.5 × 10^−4^ mM: *n* = 41). A fourth group was injected with PBS as a control (*n* = 41). Sting responsiveness was measured in response to increasing voltages during shock trials **(A)** and during placement trials in which the bees were placed in the setup without stimulation **(B)**. All three epinastine concentrations induced the same responsiveness as the PBS control both in the shock and in the placement trials, thus showing that OA does not play a significant role in sting responsiveness to a noxious stimulus.

PBS-injected bees (*n* = 41) showed a typical increase in responsiveness with increasing voltages (Roussel et al., 2009), which reached 100% at 8 V. Injection of the OA blocker epinastine did not have a significant effect on shock responsiveness (Figure 2A). All three epinastine concentrations assayed (3.5 mM: *n* = 40; 3.5 × 10^−2^ mM: *n* = 41; 3.5 × 10^−4^ mM: *n* = 41) induced the same responsiveness as the PBS control [Two-Way ANOVA: *F*_(3, 159)_ = 1.48, *p* = 0.22].

Responses to placements in the setup (Figure 2B) interspersed between shock trials remained low along the experiment and were unaffected by epinastine [*F*_(3, 159)_ = 0.48, *p* = 0.70]. Thus, neither were the bees sensitized nor did epinastine change their basal responsiveness.

### Effects of DA blocking on aversive responsiveness

**Figure 3:**
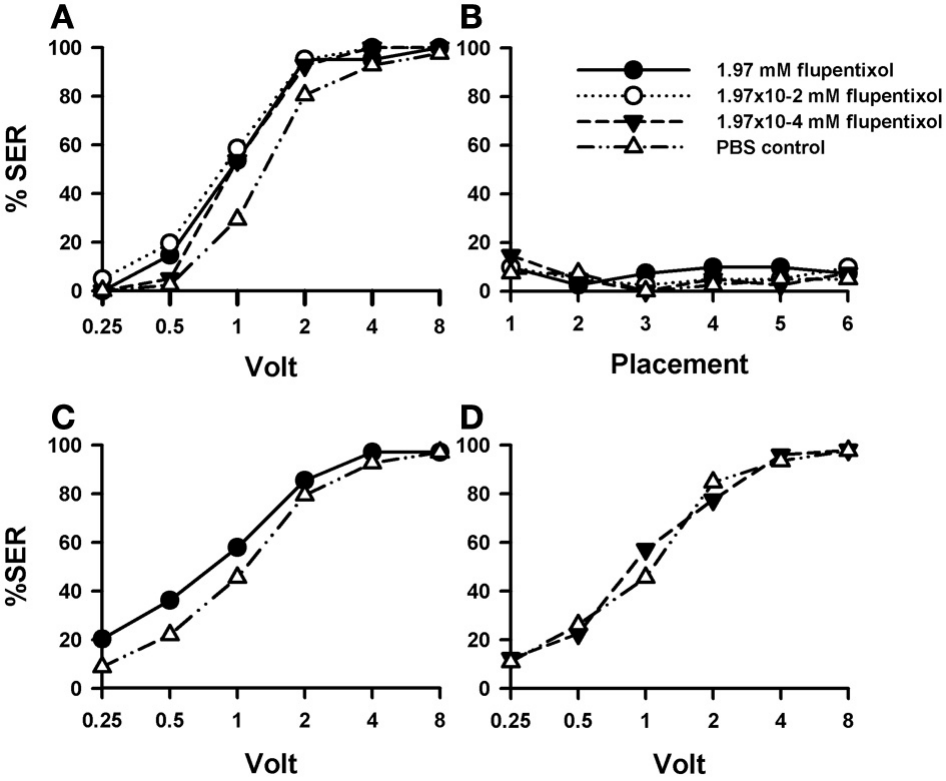
**Effects of DA blocking on aversive responsiveness.** Three different groups of bees were injected with three different concentrations of the DA antagonist flupentixol (1.97 mM: *n* = 41; 1.97 × 10^−2^ mM: *n* = 41; 1.97 × 10^−4^ mM: *n* = 41). A fourth group was injected with PBS as a control (*n* = 41). Sting responsiveness was measured in response to increasing voltages during shock trials **(A)** and during placement trials **(B)**. All three flupentixol concentrations induced an increase of responsiveness to electric shocks compared to PBS controls [*F*_(3, 160)_ = 5.46, *p* < 0.01]. No differences were found between flupentixol-injected and PBS-injected bees in the placement trials. **(C)** In a replicate of this experiment, another group of bees was injected with the highest flupentixol concentration (1.97 mM: *n* = 69) and their response to increasing voltages was measured. An increase in responsiveness to shocks with respect of the PBS control was verified (*n* = 68) [*F*_(1, 135)_ = 4.07, *p* < 0.05). Thus, DA signaling plays a significant inhibiting role in sting responsiveness to noxious stimuli as its blockade increased shock sensitivity. **(D)** In a further replicate the lowest flupentixol concentration (1.97 × 10^−4^ mM; *n* = 49) was again tested with its corresponding PBS control (*n* = 46). In this case, flupentixol did not induce an increase of responsiveness with respect to PBS-injected bees [*F*_(1, 93)_ = 0.03, *p* = 0.87]. Thus, the flupentixol concentration of 1.97 × 10^−4^ mM was certainly not excessive and the effects of higher concentrations targeted specifically dopaminergic receptors.

Injection of the DA blocker flupentixol into the bee brain induced a significant increase in shock responsiveness compared with PBS-injected bees [Figure 3A; *F*_(3, 160)_ = 5.46, *p* < 0.01]. There were no significant differences between flupentixol-injected bees [*F*_(2, 120)_ = 1.08, *p* = 0.34], thus showing that all three concentrations of this drug had the same enhancing effect. Each of the three flupentixol concentrations assayed increased responsiveness to intermediate voltages with respect of the responsiveness exhibited by PBS-injected bees [1.97 mM: *n* = 41 *F*_(1, 80)_ = 7.28, *p* < 0.01; 1.97 × 10^−2^ mM: *n* = 41 *F*_(1, 80)_ = 13.02, *p* < 0.001, and 1.97 × 10^−4^ mM; *n* = 41: *F*_(1, 80)_ = 7.07, *p* < 0.01]. Tukey *post hoc* tests showed that increases with respect to PBS-injected bees were significant for 1 V in all three concentrations (*p* < 0.001 for all three comparisons) and for 0.5 V in the intermediate concentration (1.97 × 10^−2^ mM: *p* < 0.05) but not for the other voltages despite barely non-significant results in the intermediate voltage of 2 V. Thus, by blocking the DA system, responsiveness to electric shocks of intermediate voltage was significantly increased.

Responsiveness during placement trials remained low and constant both for PBS- and flupentixol-injected bees [Figure 3B; *F*_(3, 160)_ = 0.41, *p* = 0.74] so that the neither the injection procedure nor the placement trials *per se* affected basal responsiveness.

The fact that the blocking of DA signaling increased shock responsiveness was unexpected as it had been previously found that this signaling mediates the reinforcing properties of the electric shock (Vergoz et al., 2007). Its suppression was expected to *lower* shock responsiveness. We thus decided to verify this finding. We performed a replicate of this experiment to verify the enhancing effect of flupentixol using the highest concentration previously used (1.97 mM: *n* = 69) and a PBS group as a control (*n* = 68). The results of this replicate (Figure 3C) confirmed that blocking the DA system via flupentixol injection increases shock responsiveness [*F*_(1, 135)_ = 4.07, *p* < 0.05]. Responses in placement trials remained low and unaffected by flupentixol (not shown) so that there were no differences between flupentixol- and PBS-injected bees in these trials [*F*_(1, 135)_ = 0.01, *p* = 0.97]. Finally, we performed a further replicate aimed at testing again the lowest flupentixol concentration previously used [1.97 × 10^−4^ mM]. This was necessary as in the first replicate (Figure 3A) a significant increase of responsiveness was already visible at this lowest concentration, thus raising the question of whether this effect really reflected a flupentixol blockade of dopaminergic receptors or was rather a non-specific effect due to the use of an immoderate drug concentration. As preliminary results showed that this concentration could yield non-significant effects on shock responsiveness, we measured shock responsiveness in bees injected with this flupentixol concentration (1.97 × 10^−4^ mM; *n* = 49) and with PBS (*n* = 46). Results from this replicate (Figure 3D) showed that contrarily to the highest flupentixol concentration, which consistently enhanced responsiveness in different replicates (Figures 3A,C), the lowest concentration did not induce a significant increase of responsiveness with respect to PBS-injected bees [*F*_(1, 93)_ = 0.03, *p* = 0.87]. Responses in placement trials did not differ between flupentixol- and PBS-injected bees [*F*_(1, 93)_ = 0.008, *p* = 0.93]. Thus, the flupentixol concentration of 1.97 × 10^−4^ mM was probably on the verge of significance and certainly not excessive. This ensures that, globally, the flupentixol concentrations used were moderate and targeted specifically dopaminergic receptors.

### Effects of 20E on aversive responsiveness

We then tested the effect of 20E on sting responsiveness to aversive stimulations. It has been reported that sting responsiveness is unaffected by injection of 20E (Geddes et al., 2013); yet, a single concentration was used in this study (0.312 mM) so that caution is required before generalizing this conclusion. Because 20E levels correlate inversely with aversive learning success (Geddes et al., 2013), injection of a higher concentration of 20E could impair sting responsiveness.

**Figure 4:**
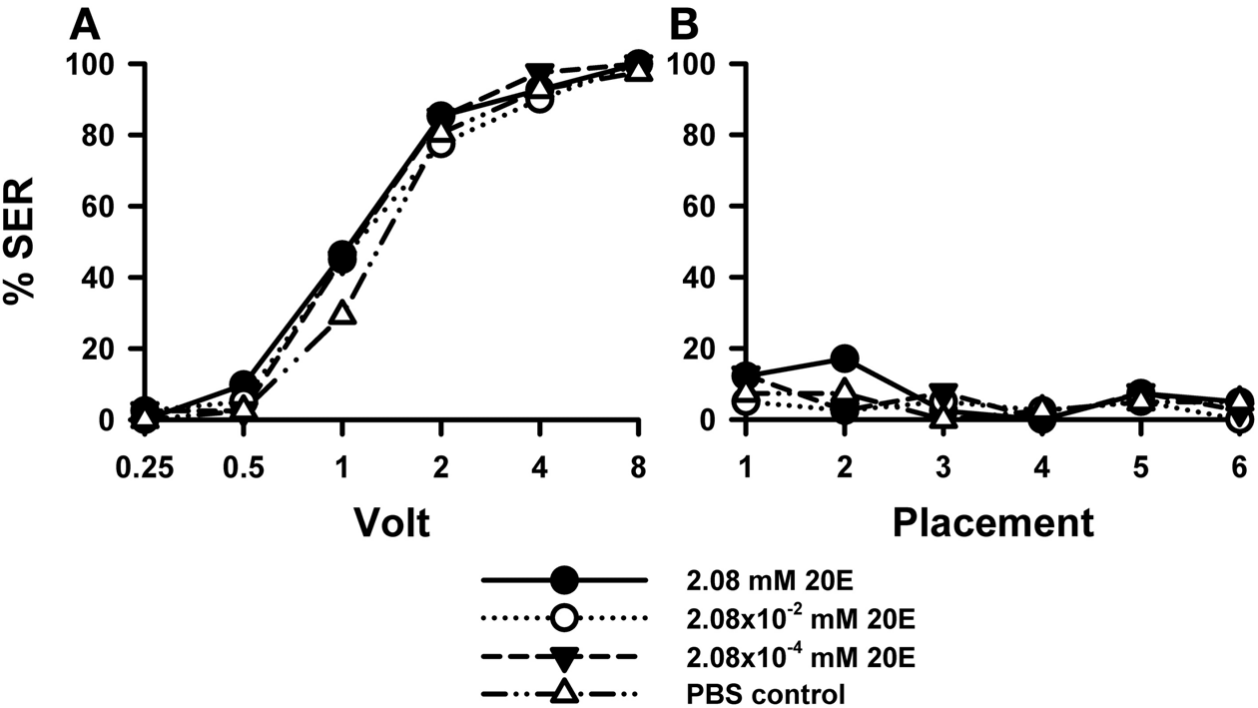
**Effects of 20E injection on aversive responsiveness.** Three different groups of bees were injected with three different concentrations of 20E (2.08 mM: *n* = 41; 2.08 × 10^−2^ mM: *n* = 40; 2.08 × 10^−4^ mM: *n* = 40). A fourth group was injected with PBS as a control (*n* = 41). Sting responsiveness was measured in response to increasing voltages during shock trials **(A)** and during placement trials **(B)**. All three 20E concentrations induced the same responsiveness as the PBS control in the shock and in the placement trials, thus showing that 20E does not play a significant role in sting responsiveness to a noxious stimulus.

Injection of 20E did not affect sting responsiveness to electric shocks (Figure 4A) compared to PBS controls [*F*_(3, 158)_ = 0.93, *p* = 0.43]. For all three 20E concentrations assayed (2.08 mM: *n* = 41, 2.08 × 10^−2^ mM: *n* = 40, 2.08 × 10^−4^ mM: *n* = 40] responses to increasing voltages augmented in a similar way as that of control bees (*n* = 41). Responses during placement trials (Figure 4B) interspersed between shock trials remained low along the experiment and were unaffected by 20E [*F*_(3, 158)_ = 0.72, *p* = 0.54]. A second replicate of this experiment (Suppl. Figure 1) yielded the same results: for all three concentrations tested the response of 20E-injected bees was the same as that of control bees both in shock [Suppl. Figure 1A: *F*_(3, 143)_ = 1.90, *p* = 0.13] and in placement trials [Suppl. Figure 1B: *F*_(3, 143)_ = 0.69, *p* = 0.55].

Thus, irrespective of the concentration of 20E used, the proportion of bees responding reflexively with sting extension to electric shocks increased in a similar way as in control bees, thus showing that 20E did not induce variations in shock sensitivity.

### Effects of 5-HT blocking on aversive responsiveness

The role of 5-HT in aversive responsiveness was studied in more detail given the lack of prior reports on the role of this biogenic amine in aversion and aggression in honey bees. Three different blockers of 5-HT signaling were used: while cyproheptadine shows potent non-competitive inhibition (Howarth et al., 2002), ketanserin and methiothepin show potent competitive inhibition in the presence of 5-HT (Howarth et al., 2002; Vleugels et al., 2013). Methiothepin acts as a non-specific antagonist of all known 5-HT receptors (Am5-HT_1A_, Am5-HT_2α_ and Am5-HT_7_) with the exception of Am5-HT_2β_ (Schlenstedt et al., 2006; Thamm et al., 2010, 2013). Ketanserin is an antagonist of Am5-HT_2β_ receptor (Thamm et al., 2013); its effect on other Am5-HT receptors is unknown. Finally, cyproheptadine antagonizes both the Am5-HT_2α_ and the Am5-HT_2β_ receptors (Thamm et al., 2013).

### Effects of 5-HT blocking by methiothepin

**Figure 5:**
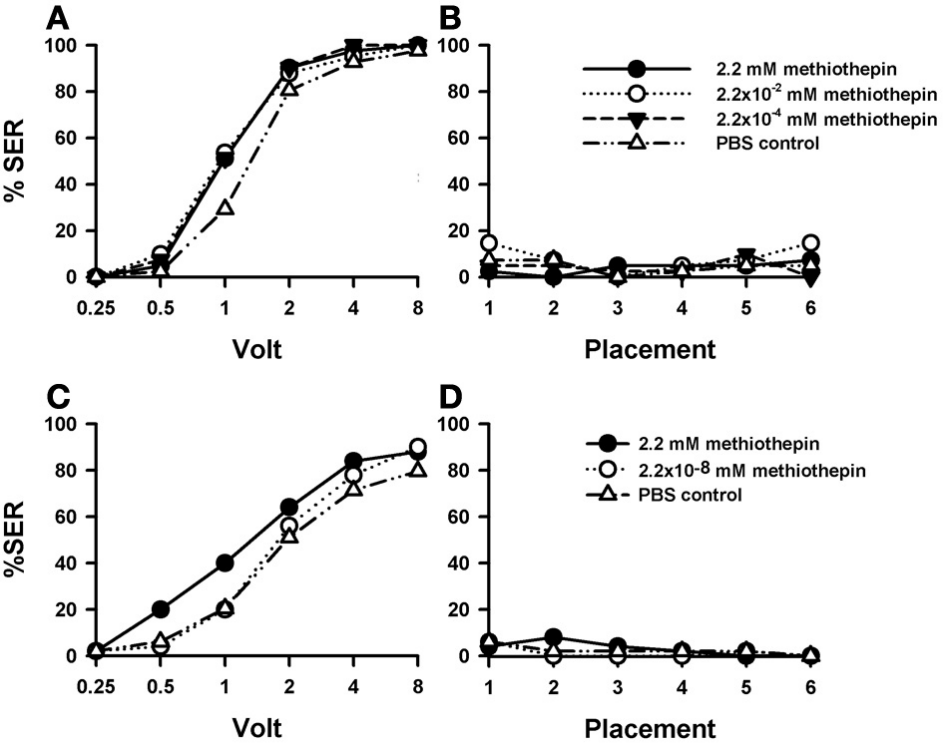
**Effects of 5-HT blocking on aversive responsiveness. (A,B)** Three different groups of bees were injected with three different concentrations of the 5-HT antagonist *methiothepin* (2.2 mM: *n* = 41; 2.2 × 10^−2^ mM: *n* = 41; 2.2 × 10^−4^ mM: *n* = 41). A fourth group was injected with PBS as a control (*n* = 41). Sting responsiveness was measured in response to increasing voltages during shock trials **(A)** and during placement trials **(B)**. Taken globally, the three methiothepin concentrations assayed induced an almost significant increase of shock responsiveness when compared to the PBS control [*F*_(3, 160)_ = 2.62, *p*= 0.052]. Yet, pairwise comparisons showed that each methiothepin concentration induced a significant increase of shock responsiveness with respect to PBS control (*p* < 0.05 for all three comparisons). There were no differences between methiothepin-injected and PBS-injected bees in the placement trials. **(C,D)** A further replicate using a lower concentration of methiothepin. Two different groups were injected with two different concentrations of methiothepin, the highest one used in the previous replicate (2.2 mM: *n* = 50) and a lower one (2.2 × 10^−8^ mM: *n* = 50). A third group was injected with PBS as a control (*n* = 49). Sting responsiveness was measured in response to increasing voltages during shock trials **(C)** and during placement trials in which the bees were placed in the setup without stimulation **(D)**. The highest methiothepin concentration induced a significant increase of responsiveness during shock trials [*F*_(1, 97)_ = 4.75, *p* = 0.032] while the lowest concentration did not [*F*_(1, 97)_ = 0.48, *p* = 0.49]. No differences were detected in the placement trials.

Injection of the 5-HT blocker methiothepin into the brain induced an increase in shock responsiveness with respect of PBS-injected bees (*n* = 41) which was close to significance [Figure 5A; *F*_(3, 160)_ = 2.62, *p*= 0.052]. There were no significant differences between methiothepin-injected bees [*F*_(2, 120)_ = 0.03, *p* = 0.96], thus showing that all three concentrations of this drug had the same enhancing effect. Nevertheless, pairwise comparisons between the PBS control and each of the methiothepin concentrations yielded a significant result in each case [2.2 mM: *n* = 41, *F*_(1, 80)_ = 4.62, *p* < 0.05; 2.2 × 10^−2^ mM: *n* = 41, *F*_(1, 80)_ = 4.32, *p* < 0.05; 2.2 × 10^−4^ mM: *n* = 41, *F*_(1, 80)_ = 5.75, *p* < 0.05]. Thus, when analyzed separately, all three concentrations increased significantly sting responsiveness to electric shock. Tukey tests showed that increases with respect to PBS-injected bees were significant for 1 V in all methiothepin concentrations (2.2 mM and 2.2 × 10^−4^ mM: *p* < 0.01, 2.2 × 10^−2^ mM: *p* < 0.001). Thus, methiothepin injections increased significantly the responsiveness to an electric shock of intermediate voltage. On the contrary, they did not affect the basal responsiveness in placement trials as SER remained low and similar to that of PBS controls [Figure 5B; *F*_(3, 160)_ = 1.58, *p* = 0.20].

A replicate of this experiment was performed in order to include a methiothepin concentration lower than those used above. This was necessary to avoid possible side effects such as a lack of specificity due to excessive drug concentration (see above “Effects of DA blocking on aversive responsiveness”). Given that in the previous replicate the effect of methiothepin already saturated at the lowest concentration (2.2 × 10^−4^ mM), we now tested the effect of methiothepin 2.2 × 10^−8^ mM (*n* = 50) to demonstrate that in our previous experiment the effects were specific and the drug was used at moderate concentrations. In parallel, the effect of the highest concentration (2.2 mM) was again tested (*n* = 50), together with the corresponding PBS control (*n* = 49). Figure 5C shows that, as in the previous replicate, the highest concentration of methiothepin induced a significant increase of responsiveness during shock trials when compared to the control [2.2 mM: *F*_(1, 97)_ = 4.75, *p* < 0.05]. On the contrary, the lowest concentration did not [2.2 × 10^−8^ mM: *F*_(1, 97)_ = 0.48, *p* = 0.49]. Neither the high nor the low methiothepin concentration affected basal responsiveness in placement trials (Figure 5D) in which SER remained low and similar to that of PBS controls [*F*_(2, 146)_ = 6.00, *p* = 0.55]. The differential effect of these two methiothepin concentrations on shock responsiveness shows that our experiments were done at reasonably moderate drugs concentrations, so that the enhancing effect induced by higher methiothepin concentrations was indeed through blockade of 5-HT receptors.

A further replicate of this experiment was performed using the highest concentration of methiothepin (2.2 mM; *n* = 67) and a corresponding PBS group (*n* = 65) to verify the enhancing effect of methiothepin on shock responsiveness (Suppl. Figure 2). The response of methiothepin-injected bees showed again an increase of responsiveness to electric shocks which, in this case, was close to significance [Suppl. Figure 2A: *F*_(1, 130)_ = 3.34, *p* = 0.07]. Placement trials also showed an increased in responsiveness in methiothepin-injected bees with respect of PBS-injected bees [Suppl. Figure 2B: *F*_(1, 130)_ = 8.30, *p* < 0.01], thus showing that methiothepin induced in this case a general, non-specific increase in responsiveness. Interestingly, this increase occurred at the begin of the experiment and vanished along placement trials [*F*_(5, 650)_ = 28.34, *p* < 0.001], thus showing a potential habituating effect.

Taken together these findings indicate that the 5-HT system plays a significant role in sting responsiveness to electric shocks and that it can even underlie general arousal and non-specific responsiveness.

### Effects of 5-HT blocking by ketanserin

**Figure 6:**
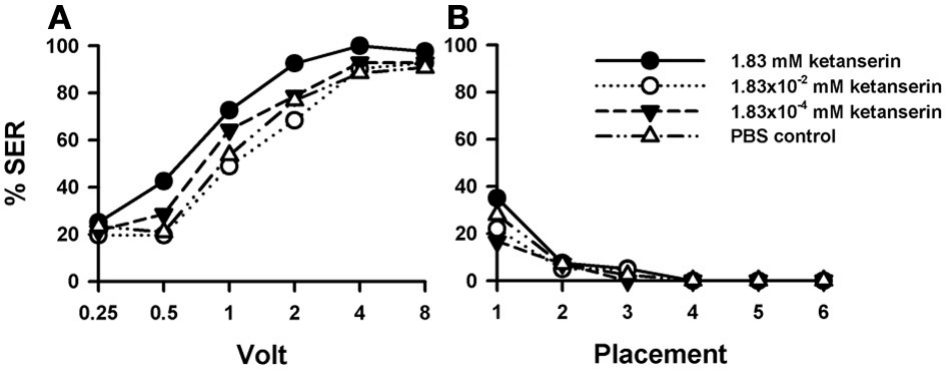
**Effects of 5-HT blocking on aversive responsiveness. (A,B)** Three different groups of bees were injected with three different concentrations of the 5-HT antagonist *ketanserin* (1.83 mM: *n* = 40; 1.83 × 10^−2^ mM: *n* = 41; 1.83 × 10^−4^ mM: *n* = 42). A fourth group was injected with PBS as a control (*n* = 43). Sting responsiveness was measured in response to a series of increasing voltages during shock trials **(A)** and during placement trials **(B)**. Only the highest ketanserin concentration increased significantly shock responsiveness with respect to the control (1.83 mM: *p* < 0.05) while the other two concentrations did not (1.83 × 10^−2^ mM and 1.83 × 10^−4^ mM: NS in both cases). No differences were detected in the placement trials.

Injection of the 5-HT blocker ketanserin induced a significant increase in shock responsiveness to electric shocks with respect of the PBS control [Figure 6A: *F*_(3, 162)_ = 2.92, *p* < 0.05]. There were significant differences between the three groups of bees injected with ketanserin [*F*_(2, 120)_ = 3.89, *p* < 0.05], as the increase in shock responsiveness was more evident for the higher ketanserin concentration (1.83 mM). This conclusion was confirmed by the pairwise comparisons between PBS controls (*n* = 43) and the three ketanserin groups: only the highest concentration increased significantly shock responsiveness [1.83 mM: *n* = 40, *F*_(1, 81)_ = 6.25, *p* < 0.05] while the other two concentrations did not [1.83 × 10^−2^ mM: *n* = 41, *F*_(1, 82)_ = 0.18, *p* = 0.67; 1.83 × 10^−4^ mM: *n* = 42, *F*_(1, 83)_ = 0.54, *p* = 0.46]. Tukey tests used to compare PBS responses and responses at the highest ketanserin concentration revealed significant differences at 1 V (*p* < 0.05).

In placement trials (Figure 6B) there was no difference between ketanserin- and PBS-injected bees [*F*_(3, 162)_ = 0.95, *p* = 0.41]. Yet, in both cases, an increase in general responsiveness was observed at the beginning of the placement trials, which vanished afterwards in successive placement trials [*F*_(5, 600)_ = 27.20, *p* < 0.001].

Thus, sting responsiveness to electric shocks was increased by the injection of ketanserin in its highest concentration. As for one of the methiothepin replicates (Suppl. Figure 2B), we observed an increase of placement responsiveness, which returned afterwards to basal levels along trials.

### Effects of 5-HT blocking by cyproheptadine

**Figure 7:**
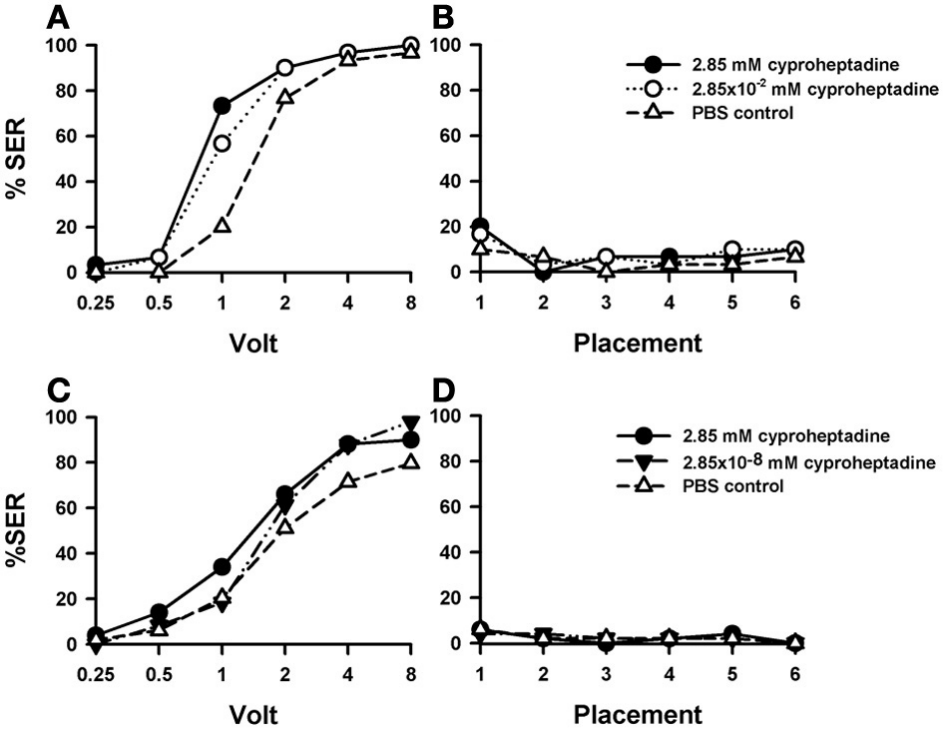
**Effects of 5-HT blocking on aversive responsiveness. (A,B)** Two different groups of bees were injected with two different concentrations of the 5-HT antagonist *cyproheptadine* (2.85 mM: *n* = 30; 2.85 × 10^−2^ mM: *n* = 30). A third group was injected with PBS as a control (*n* = 30). Sting responsiveness was measured in response to a series of increasing voltages during shock trials **(A)** and during placement trials **(B)**. Both cyproheptadine concentrations induced a significant increase of shock responsiveness when compared to PBS controls [*F*_(2, 87)_ = 7.81, *p* < 0.001] but not change of responsiveness during the placement trials. **(C,D)** A further replicate using a lower concentration of cyproheptadine. Two different groups of bees were injected with two different concentrations of cyproheptadine, the highest one used in the previous replicate (2.85 mM: *n* = 50) and a lower one (2.85 × 10^−8^ mM: *n* = 49). A third group was injected with PBS as a control (*n* = 49). Sting responsiveness was measured in response to increasing voltages during shock trials **(C)** and during placement trials in which the bees were placed in the setup without stimulation **(D)**. The highest concentration did induce a significant increase of responsiveness during shock trials [*F*_(1, 97)_ = 4.79, *p* = 0.031]. The lowest concentration did not [*F*_(1, 75)_ = 2.65, *p* = 0.11]. No differences were detected in the placement trials.

Injections of cyproheptadine induced the clearest increase in shock sensitivity [Figure 7A: *F*_(2, 87)_ = 7.81, *p* < 0.001], and thus in sting responsiveness, with respect to PBS controls (*n* = 30) and previous 5-HT antagonists (see above). There were no significant differences between the two groups of cyproheptadine-injected bees [*F*_(1, 58)_ = 0.87, *p* = 0.35], thus showing that both concentrations (2.85 mM: *n* = 30; 2.85 × 10^−2^ mM: *n* = 30) had the same enhancing effect. Indeed, each cyproheptadine concentration taken separately increased significantly sting responsiveness with respect of PBS controls [2.85 mM: *F*_(1, 58)_ = 13.80, *p* < 0.001; 2.85 × 10^−2^ mM: *F*_(1, 58)_ = 8.12, *p* < 0.01]. Tukey tests comparing PBS responses and responses of the two cyproheptadine concentrations revealed significant differences at 1 and 2 V in both cases (*p* < 0.001).

Responses during placement trials (Figure 7B) interspersed between shock trials remained low along the experiment and were unaffected by cyproheptadine [*F*_(2, 87)_ = 0.50, *p* = 0.61]. Thus, blocking 5-HT signaling by cyproheptadine determined a significant increase in shock but not in placement responsiveness.

A replicate of this experiment was performed in order to include a cyproheptadine concentration lower than those used above. Given that in the previous replicate the effect of cyproheptadine already saturated at the lowest concentration tested (2.85 × 10^−2^ mM), we now tested the effect of cyproheptadine 2.85 × 10^−8^ mM (*n* = 49) to demonstrate that in our previous experiment this drug was used at moderate concentrations so that cyproheptadine effects were specific. In parallel, the effect of the highest concentration (2.85 mM) was again tested (*n* = 50), together with the corresponding PBS control (*n* = 49). Figure 7C shows that, as in the previous replicate, the highest concentration of cyproheptadine induced a significant increase of responsiveness during shock trials when compared to the control [2.85 mM: *F*_(1, 97)_ = 4.79, *p* < 0.05] while the lowest concentration did not [*F*_(1, 75)_ = 2.65, *p* = 0.11]. Neither the high nor the low cyproheptadine concentration affected basal responsiveness in placement trials (Figure 7D) in which SER remained low and similar to that of PBS controls [*F*_(2, 145)_ = 0.0005, *p* = 0.99]. The differential effect of these two cyproheptadine concentrations on shock responsiveness shows that our experiments were done at reasonably moderate drugs concentrations, so that the enhancing effect induced by higher methiothepin concentrations was indeed through blockade of 5-HT receptors.

## Discussion

Our study provides the first neuropharmacological dissection of the neurotransmitter systems underlying the central control of sting responsiveness to noxious stimuli in honey bees. By injecting pharmacological antagonists into the bee brain, we determined the effect of blocking different forms of neurotransmission on shock responsiveness. We found that both OA and 20E are dispensable for shock responsiveness while DA and 5-HT act as repressors of sting responsiveness; antagonists of these two biogenic amines induce an increase in shock responsiveness to shocks of intermediate voltage.

### Octopamine and aversive responsiveness

OA blocking through epinastine did not affect sting responsiveness to electric shock. Injection of three different concentrations of the OA antagonist epinastine into the bee brain did not induce any change in SER thus indicating that this biogenic amine is not involved in the central control of this reflexive response. Epinastine was chosen due to its high specificity and affinity to OA receptors (Roeder et al., 1998). Mianserin, another drug previously used as OA antagonist in the bee, was avoided due to its side-effects on the serotonergic system (Fernández et al., 2012). Epinastine blocks specifically AmOA1, the only OA receptor identified so far in the bee (Grohmann et al., 2003; Farooqui et al., 2004). This OA receptor is thus dispensable for shock responsiveness.

Isolated abdominal preparation have been used to show that OA reduces the level of rhythmic neuromuscular activity during stimulated stinging response trials, but does not alter the activity in pre-stimulation baseline trials or post-stimulation recovery trials. Local applications of OA at the level of the abdominal preparations showed that OA also potentiates SER and this potentiation persisted for at least 3h (Burrell and Smith, 1995). Yet, the same isolated abdominal preparations showed no differences between castes in sting responsiveness (Burrell and Smith, 1994) although it is clear that such differences exist (Lenoir et al., 2006; Roussel et al., 2009). It was thus concluded that “*any effect of caste must arise in more anterior ganglia and/or in the brain*” (Burrell and Smith, 1994). In our case, we conclude that the lack of effect of epinastine in our *in toto* preparation shows that the abdominal effects of OA are under central control.

The lack of effect of OA antagonism on shock responsiveness is in agreement with the notion of modularity of appetitive vs. aversive behaviors (Roussel et al., 2009; Tedjakumala and Giurfa, 2013). In this scheme, OA is predominantly associated with appetitive behavior: OA is crucial for appetitive responsiveness as feeding or injection of both OA and tyramine, an OA precursor, significantly increase PER to sucrose stimulation (Scheiner et al., 2002). Also, in appetitive olfactory PER conditioning, OA is said to mediate the reinforcing properties of sucrose reward (Hammer, 1993; Hammer and Menzel, 1998; Farooqui et al., 2003). Therefore, pairing an odor with injections of OA in the bee brain leads to olfactory learning in harnessed bees, which exhibit afterwards PER to this odor (Hammer and Menzel, 1998). In cricket visual and olfactory learning, pharmacological blocking of OA receptors impairs the acquisition of appetitive but not aversive learning (Unoki et al., 2005, 2006). In *Drosophila* mutants that have the biosynthetic pathway to OA blocked, both learning of an odor-sucrose association and memory retention 3 min after conditioning are impaired (Schwaerzel et al., 2003). These mutants can, however, learn and memorize an aversive olfactory discrimination, in which they have to avoid an odorant previously paired with an electric shock (Schwaerzel et al., 2003). Recently, the exclusiveness of OA neurotransmission for appetitive reinforcement signaling has been reconsidered in the fruit fly where a group of dopamine neurons was found to signal sugar reward to the mushroom bodies, the site where appetitive memory traces are formed (Liu et al., 2012). These DA neurons are selectively required for the reinforcing property of, but not a reflexive response to, the sugar stimulus, which is mediated by OA. Thus, OA-dependent memory formation requires signaling through DA neurons (Burke et al., 2012). These experiments indicates that sweet taste engages a distributed OA signal that reinforces memory through discrete subsets of mushroom-body-targeted DA neurons (Burke et al., 2012). Furthermore, OA signaling also intervenes in the consolidation of aversive, intermediate-term memory in *Drosophila* (Wu et al., 2013). Following odor-shock learning, the anterior paired lateral (APL) neurons release OA to the α' and β ' Kenyon cells of the mushroom bodies and this signal is necessary for the consolidation of anesthesia resistant memory, a component of intermediate term memory retrievable 3 h after conditioning.

### Dopamine and aversive responsiveness

Flupentixol was chosen as antagonist of the dopaminergic system due to its high binding affinity to D1- as well as D2-like receptors (Kokay and Mercer, 1996). Among various dopaminergic antagonists assayed in olfactory SER conditioning, it proved to be highly effective to impair olfactory acquisition and mid-term retention (Vergoz et al., 2007), thus indicating that dopaminergic signaling underlies the aversive reinforcement properties of the electric shock. Other dopaminergic antagonists such as fluphenazine have been assayed on aversive olfactory conditioning and had less effect on behavioral performances (Vergoz et al., 2007).

In the present work, dopaminergic blocking through flupentixol induced an increase of shock responsiveness for low/intermediate voltages (0.5–1 V), thus reflecting an enhancement in shock sensitivity. At higher concentrations no differences between flupentixol and PBS-injected bees was found, probably because of a ceiling effect. The increase of shock sensitivity at lower voltages was observed for different concentrations of flupentixol and in different replicates of this experiment, thus showing that the effect was robust and repeatable. The result thus indicates that DA acts as a depressor of sting responsiveness to electric shocks so that when its effect is antagonized, responsiveness increases.

This results is consistent with those of studies in which the effect of DA on sucrose responsiveness was analyzed (Scheiner et al., 2002). DA *decreases* sucrose responsiveness when injected into the thorax. Also, injection or feeding of the DA receptor agonist 2-amino-6,7-dihydroxy-1,2,3,4-tetrahydronaphthalene (6,7-ADTN) reduces sucrose responsiveness significantly (Scheiner et al., 2002). Although we did not test 6,7-ADTN, it can be predicted that injection of this DA receptor agonist should also decrease shock responsiveness. In olfactory PER conditioning, injection of DA into the antennal lobes reduces significantly olfactory retention both after one and three conditioning trials (Macmillan and Mercer, 1987). DA seems, therefore, to play a depressing role in a series of appetitive and aversive responses.

Yet, a different role for DA was suggested based on protocols of aversive conditioning in bees, crickets and flies. Besides the above-mentioned fact that a subset of DA neurons convey an appetitive reinforcement signal to MBs, the role of the dopaminergic system in insect learning has been related to aversive-reinforcement signaling in the insect brain. In crickets, pharmacological blocking leads to an impairment of visual and olfactory aversive learning (Unoki et al., 2005, 2006). In adult fruit flies, blockade of DA neurons impairs olfactory aversive learning (Schwaerzel et al., 2003); activation of a specific subset of DA neurons (distinct from that conveying appetitive signals, see above) in mutant flies substitutes for shock reinforcement in aversive olfactory conditioning (Claridge-Chang et al., 2009; Aso et al., 2010, 2012); similar results were obtained in *Drosophila* larvae where activation of DA neurons contingent to odor presentation results in odor avoidance (Schroll et al., 2006), thus showing that a specific subset of DA neurons substitute for aversive reinforcement in aversive learning. As mentioned before, in the honey bee, a similar conclusion was originally reached in aversive olfactory SER conditioning (Vergoz et al., 2007): in this case, injection of the DA antagonists flupentixol into the bee brain suppresses the capacity to learn and retrieve odor-shock associations (Vergoz et al., 2007), thus suggesting that, in this case too, DA mediates aversive-reinforcement signaling necessary for aversive learning. Importantly, specific controls showed in some (but not all) of these studies that DA blockade or activation did neither affect motor responses nor sensory perception, so that in the framework of aversive conditioning DA does not down-regulate behavior in a non-specific way; instead, it acts specifically as an aversive reinforcement signal.

How is it then possible to reconcile these two functions? If DA blockade facilitates behavior owing to the general depressor effect of this biogenic amine, why were motor and sensory functions unaffected in the conditioning protocols discussed above despite DA blockade? If DA signaling mediates the aversive reinforcing properties of the electric shock and its blockade impairs aversive learning, why does its blockade in shock responsiveness experiments (this work) *enhance* shock sensitivity? This result was unexpected as we assumed, based on the previous work on olfactory SER conditioning (Vergoz et al., 2007), that blocking the dopaminergic system would diminish the aversive reinforcement properties of the electric shock, thus decreasing shock responsiveness. Importantly, in our work as in that on olfactory SER conditioning (Vergoz et al., 2007) the same antagonist (flupentixol), the same injection site (ocellar tract) and dose (1.97 mM) were used, so that functional differences are not due to these experimental variables.

A possible explanation for this dual function is to assume the existence of, at least, two different classes of dopaminergic neurons mediating different functions: one acting as a *general gain control system*, with the specific role of down-regulating responsiveness and another acting as *instructive* neurons in aversive associative learning which mediates aversive US signaling. Owing to these different functions, their brain targets could be different. While the first class would exhibit extensive and broad branching within the entire brain in order to be able to modulate different motivational components (appetitive, aversive) and sensory modalities (olfactory, visual gustatory, etc.), the second class would exhibit a specific connectivity with respect to CS processing circuits (olfactory, visual) in order to facilitate CS-US associations and provide instructive (i.e., valence) information to the targeted CS circuit (Giurfa, 2006). These two classes may also differ in terms of the dopaminergic receptors they express.

In vertebrates, dopaminergic receptors are generally classified in two main families, the D1-like and D2-like receptors (Jaber et al., 1996; Neve et al., 2004). Activation of the D1-like family is coupled to increases in cAMP concentration and is typically excitatory, while D2-like activation reduces cAMP and is typically inhibitory. In the honey bee, three different DA receptors have been identified: AmDOP1 (Blenau et al., 1998), AmDOP2 (Humphries et al., 2003) and AmDOP3 (Beggs et al., 2005). AmDOP1 and AmDOP3 have been related to the vertebrate D1-like and D2-like family of dopamine receptors, respectively (Blenau et al., 1998; Beggs et al., 2005). AmDOP2 appears to be more closely related to invertebrate OA receptors but it has been referred to as a “D1-like receptor” because it up-regulates cAMP (Humphries et al., 2003). In the case of olfactory SER conditioning, DA blockade by means of vertebrate D1-like and D2-like receptor blockers SCH23390 and spiperone, respectively, yielded different results: while SCH23390 did not impair olfactory SER conditioning, spiperone significantly impaired acquisition and retention, thus suggesting that D1-like and D2-like DA receptors contribute differently to the signaling of US reinforcement by the instructive DA neurons (Vergoz et al., 2007). In addition, the fact that 20E (see below) impairs olfactory SER conditioning, thus acting on the instructive DA neurons, but leaves intact shock responsiveness to electric shock (Geddes et al., 2013) reaffirms the heterogeneity of the DA signaling mechanisms in the bee brain.

We suggest that the first class of DA neurons, acting as general gain control system, could mediate responding adaptively to appropriate stimuli in the insect's environment. It may therefore mediate attentional processes in which perception is focused on one stimulus (or group of related stimuli), while filtering out other simultaneous stimuli that are less relevant at any moment (Posner et al., 1980). Attentional processes, similar to those described in vertebrates, can also be identified in insects (Dyer and Chittka, 2004; Giurfa, 2004; Miller et al., 2011; Van Swinderen, 2011; Van Swinderen and Andretic, 2011) and, in the case of *Drosophila*, a neural correlate of such processes is a transient increase in a 20–30 Hz local field-potential recorded in a region of the brain called the medial protocerebrum (Van Swinderen and Greenspan, 2003). Current views relate DA levels in the insect brain with arousal levels (Van Swinderen and Andretic, 2011). Transient attenuation of DA release in fly mutants attenuates the 20–30 Hz responsiveness to the object to be attended and oral delivery of methamphetamine, which increases DA release, rescues this responsiveness (Andretic et al., 2005). Thus, gain-control DA neurons may modulate selective attention in the insect brain, acting on a series of nervous circuits underlying different forms of sensory-motor performances.

Different classes of dopaminergic neurons have been identified in the fruit fly which mediate appetitive (Burke et al., 2012; Liu et al., 2012) and aversive (Aso et al., 2012) reinforcing functions. Yet, suppressing DA signaling in mutants does not affect sensitivity to electric shocks with respect to wild-type flies (Riemensperger et al., 2011). This result does not invalidate our findings as in the fruit fly experiments, flies were tested in groups and not individually, and were subjected to a single voltage (60 V) during one minute so that no sensitivity curves were established. Appropriate behavioral measurements should show whether suppression of DA signaling does indeed leave shock sensitivity unaffected in fruit flies as claimed (Riemensperger et al., 2011), or whether it increases sensitivity to voltages lower than the one tested, consistently with our findings. In any case, we posit that besides the instructive category of DA neurons available in bees and flies, a different class of dopaminergic neurons exist which provide a down-regulating control of responsiveness upon perception of potentially noxious stimulation.

### 20E and aversive responsiveness

20-hydroxyecdisone (20E) is a metabolite of the steroid hormone ecdysone, which intervenes in insect development and reproduction (Riddiford et al., 2000). This ecdysteroid impairs aversive but not appetitive conditioning in bees (Geddes et al., 2013). Two-day old bees are deficient in olfactory SER learning (Geddes et al., 2013) in agreement with higher titers of ecdysteroids occurring at this age (Hartfelder et al., 2002). This impairment seems to be achieved in part via the dopamine/ecdysone receptor gene AmGPCR19. Exogenous 20E injection determines both a reduction in AmGPCR19 levels 3 h after injection and a decrease in aversive learning performances of adult (6-day old) bees (Geddes et al., 2013). The same 20E injection does not modify the levels of the three dopaminergic receptors known in the bee, AmDOP1, AmDOP2, and AmDOP3, 3 h after injection (McQuillan, 2013). Taken together these results indicate that at this delay the decrement of aversive learning induced by 20E occurs via AmGPCR19 and not via the AmDOP receptors.

Injection of 20E does not affect shock responsiveness to electric shock when measured 30 min (this work) or 3 h after injection (Geddes et al., 2013), and with an extended range of 20E concentrations (this work). Given that neither up- nor down-regulation of AmDOP receptors occurs 3 h after 20E injection (see above), no variation should also be expected after our shorter injection delay of 30 min. Thus, while the decrement in olfactory SER conditioning induced by 20E occurs via a decrease in AmGPCR19, we suggest that the lack of effect of 20E on shock responsiveness may be related to its lack of effect on AmDOP receptors.

The fact that 20E injection did not affect shock responsiveness indicates that the decrement in olfactory SER conditioning induced by this ecdysteroid is not due to a loss of US sensitivity. 20E could then exert a negative effect on the other components of this associative learning: it may reduce olfactory perception and/or impair the associability of CS and US pathways. Following our suggestion concerning the existence of at least two classes of dopaminergic neurons (see above), we suggest that the negative effect of 20E on aversive learning is mediated by the instructive neurons specifically involved in aversive associative learning, but not by the gain-control dopaminergic neurons. Accordingly, flupentixol impairs aversive olfactory learning (Vergoz et al., 2007) and modifies shock responsiveness [this work], whereas 20E triggers a different side-effect, impairing the learning, but not the perception to shock stimuli (Geddes et al., 2013). This suggests that flupentixol and 20E may bind/block different DA receptors and even trigger different signal cascades.

### 5-HT and aversive responsiveness

Three different blockers of 5-HT signaling were used in our work. The clearest effects on SER were obtained with cyproheptadine, which shows potent non-competitive inhibition in the presence of 5-HT (Howarth et al., 2002; Vleugels et al., 2013) and antagonizes both the Am5-HT_2α_ and the Am5-HT_2β_ receptors (Thamm et al., 2013). In this case, the two higher cyproheptadine concentrations assayed (2.85 mM and 2.85 × 10^−2^ mM) increased significantly shock sensitivity at intermediate voltages but not placement responsiveness.

Methiothepin acts as a competitive inhibitor in the presence of 5-HT, and antagonizes in a non-specific way all known 5-HT receptors (Am5-HT_1A_, Am5-HT_2α_, and Am5-HT_7_) with the exception of Am5-HT_2β_ (Schlenstedt et al., 2006; Thamm et al., 2010, 2013). Injections of this drug also increased significantly the responsiveness to electric shocks of intermediate voltage but to a lower extent than cyproheptadine. Global comparisons between responses to the methiothepin concentrations and PBS responses in all three replicates yielded barely non-significant results (*p* = 0.052, *p* = 0.07, and *p* = 0.06). Yet, pairwise comparisons between single-dose responses and PBS responses were significant in three out of four cases. In one of the four cases, methiothepin induced a general, non-specific increase in responsiveness visible at the begin of the placement trials. SER returned to basal levels along consecutive placement trials thus showing that the increased excitability following 5-HT blockade was reduced probably via habituation processes.

Finally, ketanserin is a competitive antagonist of Am5-HT_2β_ receptor (Thamm et al., 2013). Only the highest concentration of this drug increased significantly shock responsiveness with respect to PBS controls. In this case, increase of responsiveness in the first placement trial was present both in ketanserin groups and in PBS controls so that it cannot be attributed to 5-HT inhibition.

Taken together, our results provide the first analysis of the role of 5-HT in aversive responsiveness in honey bees. Injection of three different 5-HT antagonists increased to different extents shock responsiveness to intermediate voltages and in some cases, to placement trials. These results indicate that the serotonergic system acts as a depressor of aversive responsiveness and probably of a broader spectrum of behaviors. Since clearer increases in shock sensitivity were observed with cyproheptadine, it may be suggested that inhibition of general responsiveness by 5-HT requires both Am5-HT_2α_ and Am5-HT_2β_ receptors. When only one of these receptors is targeted, as seems to be the case for methiothepin (Am5-HT_2α_) and ketanserin (Am5-HT_2β_) increases in responsiveness are still visible but to a lower extent, thus suggesting an additive effect of 5-HT neurotransmission via these two receptors.

The notion of 5-HT mediating a general inhibitory system is supported by results obtained in appetitive olfactory PER conditioning. In this framework, injection of 5-HT impairs the acquisition and retrieval of olfactory memories (Mercer and Menzel, 1982; Bicker and Menzel, 1989; Menzel et al., 1999). An inhibitory role of 5-HT signaling was also found in a variant of PER conditioning used to study latent inhibition, a decrement in learning performance which results from the non-reinforced preexposure of the odor to be conditioned (Fernández et al., 2012). In this case, blockade of 5-HT by injection of ketanserine and methysergide suppresses latent inhibition and rescues learning of the pre-exposed odor. It was thus suggested that latent inhibition could be the consequence of increased levels of 5-HT, resulting from repeated unrewarded CS exposure (Fernández et al., 2012). Higher levels of 5-HT would determine an inhibitory state (or a state of reduced excitability) and would thus impair CS-US associations.

5-HT neurotransmission would thus intervene in the modulation of a broad spectrum of behaviors, acting as a general gain-control system facilitating behavioral inhibition. Serotonergic neurons in the optic ganglia can modulate visual responses such as the motion-sensitive visual antennal reflex, a typical direction specific antennal response to a stripe pattern moving up- and downward (Erber and Kloppenburg, 1995; Kloppenburg and Erber, 1995). Consistently with our hypothesis, 5-HT application into the ipsilateral lamina, lobula, and medulla, the main visual areas of the insect brain, leads to an immediate and long lasting (at least 30 min) decrease of the reflex when the ipsilateral compound eye is stimulated. In some cases, the response to stimulation of the contralateral eye is also reduced (Erber and Kloppenburg, 1995). Accordingly, 5-HT reduces background activity as well as responses to moving stripe patterns by motion-sensitive lobula neurons. The amplitudes of lobula field potentials evoked by moving stripe patterns are also reduced by application of 5-HT. Phototactic responsiveness is also strongly reduced by 5-HT but can be rescued by feeding bees a mixture of 5-HT and the Am5-HT_1A_ receptor antagonist prazosin over a 2-day period (Thamm et al., 2010).

All in all, the picture emerging from our and other studies is one in which 5-HT may allow responding adaptively to relevant stimuli of different valence (appetitive, aversive) and sensory modalities (visual, olfactory) by suppressing responses to irrelevant, non-predictive stimuli. Together with DA (see above), 5-HT may thus play an essential role in attentional processes, allowing an insect to cope efficiently with its environment.

### Biogenic amines and task division in the hive

Task division in a social insect colony is a fundamental factor for sociality (Wilson, 1971). The *response-threshold model* has been proposed to explain the division of labor in social insects. It posits that differences in sensitivity to external stimuli exist between individuals and that individuals highly sensitive to a given stimulus are prospective candidates for becoming specialized in tasks involving such a stimulus (Page and Erber, 2002). Related sensitivities to stimuli usually encountered in an appetitive context can be grouped in a “foraging behavior (or appetitive) syndrome” (Page et al., 2006), defined as a set of correlated behaviors reflecting between-individual consistency in behavior across multiple foraging situations (Sih et al., 2004). Similarly, sensitivities to stimuli usually encountered in an aversive/defensive context can define an “aversive syndrome” (Roussel et al., 2009; Tedjakumala and Giurfa, 2013). Biogenic amines may play an essential role for such specializations, modulating an individual's responsiveness to specific stimuli (Scheiner et al., 2004). For instance, OA and tyramine facilitate appetitive responses while DA inhibits appetitive aversive responses so that behavioral syndromes may be defined at the individual level through the fine balance between amines mediating appetitive and aversive responses.

In this scenario we propose that it is worth distinguishing between two different involvements of biogenic amines: on one hand some of them (OA, DA) may act as instructive signals in associative circuits attributing specific valences to stimuli to be learned (OA: appetitive; DA: aversive), and, on the other hand, they may provide global gain control systems facilitating behavioral responses through a decrease of responsiveness thresholds (OA) or, on the contrary, inhibiting such responses (DA and 5-HT) thereby determining more focused and appropriate stimulus responses. In such scenario, attentional control may be particularly relevant for the division of labor. Further studies should determine if and how response-threshold models need to incorporate such control mechanisms and whether biogenic amines such as 5-HT play a relevant role for task specialization.

## Conflict of interest statement

The authors declare that the research was conducted in the absence of any commercial or financial relationships that could be construed as a potential conflict of interest.
