# Supplementary material for: Pharmacological modulation of aversive responsiveness in honey bees
Source: Front Behav Neurosci. 2014 Jan 7;7:221. doi: 10.3389/fnbeh.2013.00221 (PMC3882874; doi:10.3389/fnbeh.2013.00221)
Supplement: Supplementary file 1 [file DataSheet1.DOCX]

**Supplementary Figure 1:** Effects of blocking by 20E on aversive responsiveness. Three different groups of bees were injected with three different concentrations of 20E (2.08 mM: n = 35; 2.08x10^-2^ mM: n = 37; 2.08x10^-4^ mM: n = 37). A fourth group was injected with PBS as a control (n = 38). Sting responsiveness was measured in response to increasing voltages during shock trials (A) and during placement trials in which the bees were placed in the setup without stimulation (B). All three 20E concentrations assayed induced the same responsiveness as the PBS control in the shock and in the placement trials, showing that 20E does not play a significant role in sting responsiveness to a noxious stimulus.

**A**

**B**
