# Supplementary material for: Pharmacological modulation of aversive responsiveness in honey bees
Source: Front Behav Neurosci. 2014 Jan 7;7:221. doi: 10.3389/fnbeh.2013.00221 (PMC3882874; doi:10.3389/fnbeh.2013.00221)
Supplement: Supplementary file 2 [file DataSheet2.DOCX]

**Supplementary Figure 2:** Effects of 5-HT blocking on aversive responsiveness. A group of bees was injected with the highest different concentrations of the 5-HT antagonist methiothepin (2.2 mM: n = 67). Another group was injected with PBS as a control (n = 65). Sting responsiveness was measured in response to a series of increasing voltages during shock trials (A) and during placement trials in which the bees were placed in the setup without stimulation (B). The highest methiothepin concentration induced an almost significant increase of responsiveness to the electric shocks compared to the PBS controls (F_1,130_ = 3.34, p = 0.07) and a significant increase of non-specific responsiveness in placement trials (F_1,130_ = 8.30, p < 0.01). These results show that 5-HT inhibits sting responsiveness to a noxious stimulus and non-specific responsiveness as its blockade through methiothepin increases both forms of responsiveness.

**B**

**A**
